# Supplementary material for: Optimizing Performance and Satisfaction in Matching and Movement Tasks in Virtual Reality with Interventions Using the Data Visualization Literacy Framework
Source: Front Virtual Real. Author manuscript; Available in PMC 2026 Jun 2. (PMC13225873; doi:10.3389/frvir.2021.727344)
Supplement: Optimizing Performance and Satisfaction in Matching and Movement Tasks in VR [file NIHMS2171915-supplement-Optimizing_Performance_and_Satisfaction_in_Matching_and_Movement_Tasks_in_VR.docx]

Supplementary Material

# Ethics Statement

The research described in this paper was approved by the Institutional Review Board at Indiana University under protocol numbers 1910331127 (RUI VR) and 1911941428 (Luddy VR). Study information sheets for both studies can be found in a GitHub repository at <https://github.com/cns-iu/optimizing-performance-in-VR-using-DVL-FW/tree/main/irb>.

# Supplementary Text for RUI VR

This GitHub repository contains relevant materials that did not make it into the paper given constraints around word count and the number of figures and tables allowed: <https://github.com/cns-iu/optimizing-performance-in-VR-using-DVL-FW>

It also contains C# scripts showing how we implemented interactions for the visualizations in the Reflective phases of the two VR studies.

## Unity Projects

To ensure reproducibility of our results, and to enable all readers to replicate the steps in our user study, we made the Unity (Unity Technologies, 2021) projects for each study available on GitHub. Below are the links to each GitHub repository:

<https://github.com/cns-iu/luddy_vr_unity_project>
<https://github.com/cns-iu/rui_vr_reflective_vr_project>
<https://github.com/cns-iu/rui_vr_user_study>

## Study design

The design for the RUI VR study is explained in more depth in an existing publication that focuses on comparing accuracy, completion time, and satisfaction between the three setups in the control cohort only. A paper describing the study design and results has been published recently (Bueckle et al., 2021a).


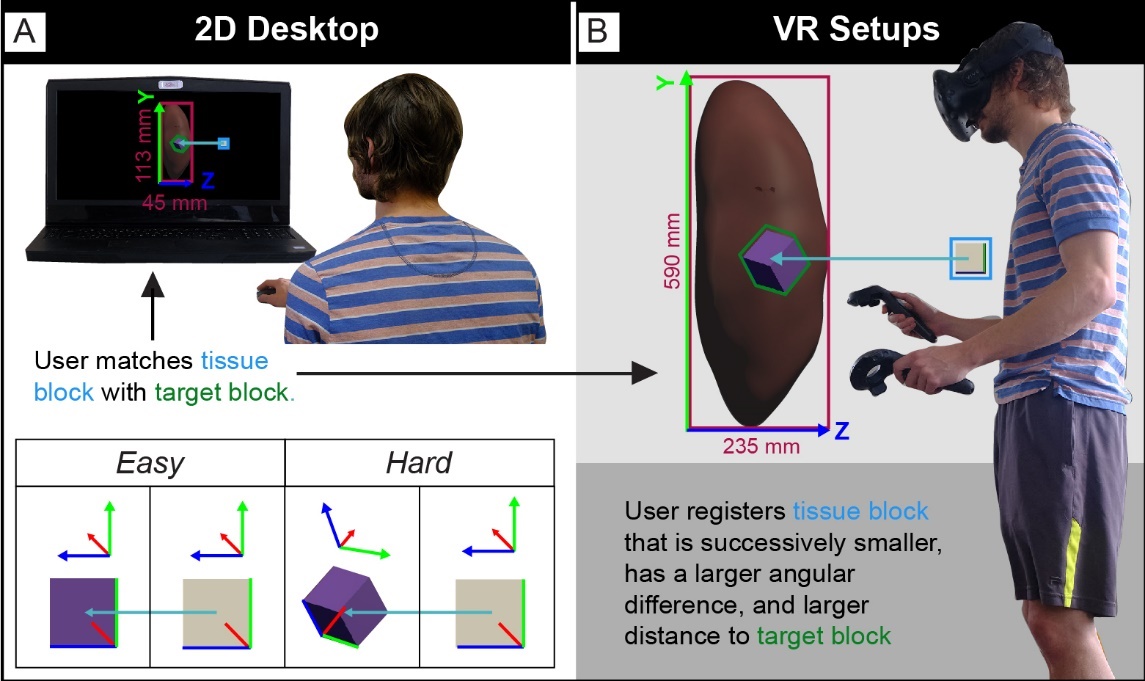


Supplementary Figure 1. The task setup in our user study: Reference organ with target block indicated (purple) and tissue block (white) to be registered into the target block. The light blue arrow indicates block centroid (mid-point) distance. Task difficulty increases as the tissue blocks get smaller, block rotation increases, and distance between the blocks increases. (A) 2D Desktop setup. (B) The two VR setups (Bueckle et al., 2021a).


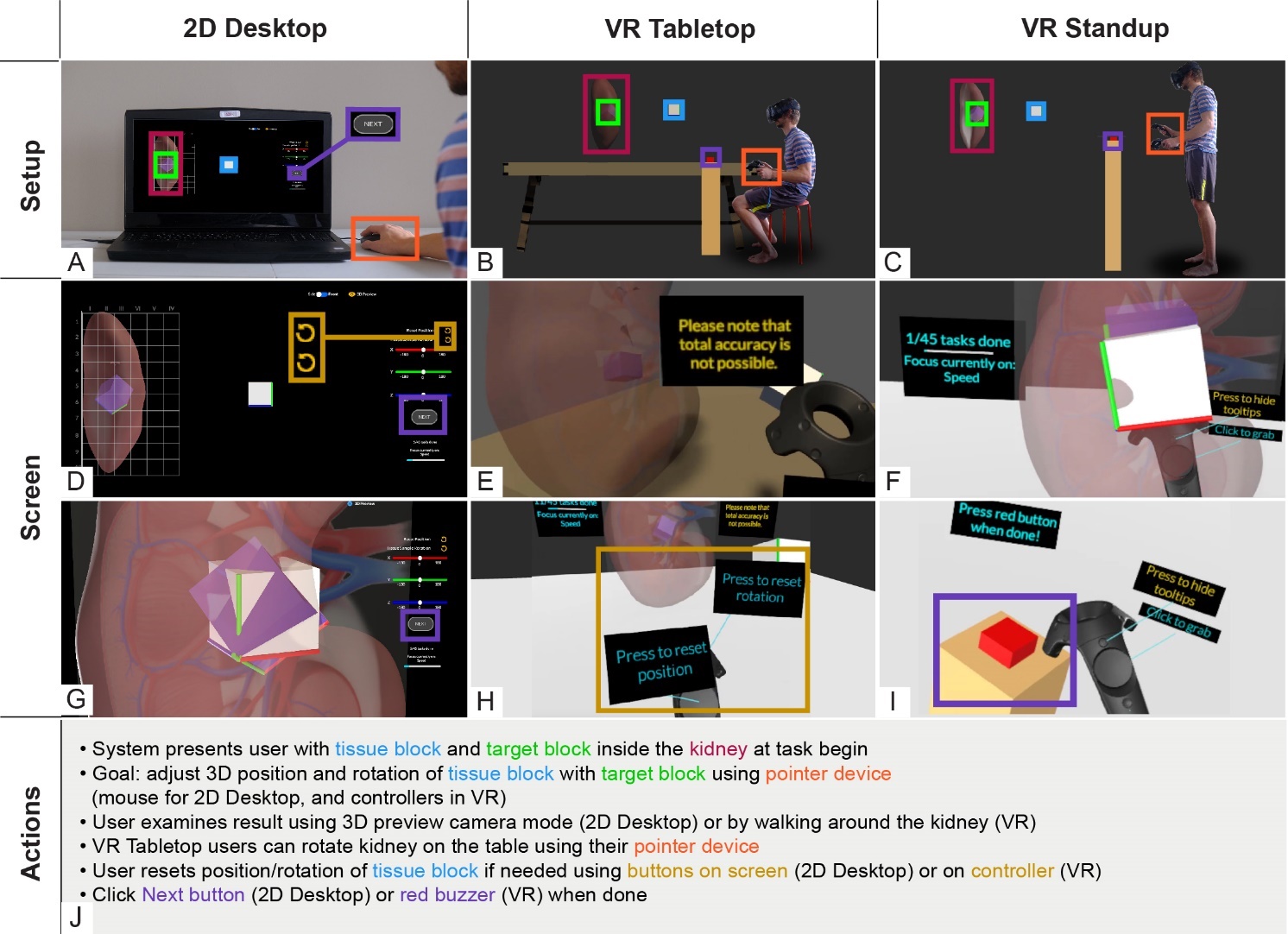


Supplementary Figure 2. Setup, screen, and actions for 2D Desktop, VR Tabletop, and VR Standup (Bueckle et al., 2021a). (A-C) Three RUI setups with a human subject. (D-I) screenshots of the user interface. (J) Required actions. The tissue block is outlined in blue, the target block in green, and the kidney—providing context and domain relevance—in pink. Tasks are submitted by selecting the purple NEXT/red button. The user could reset the position or rotation of the tissue block by pressing the corresponding yellow-brown virtual (2D Desktop) and physical buttons (VR).

$Lerp=\left\{ \begin{aligned} a, \\ b, \\ a+\left( b-a \right)*t \end{aligned} \right.\left. \begin{aligned} , if t \leq0 \\ , if t \geq1 \\ , if 0 <t< 1 \end{aligned} \right\}$

**Supplementary Equation 1.** Formula to compute task difficulty.


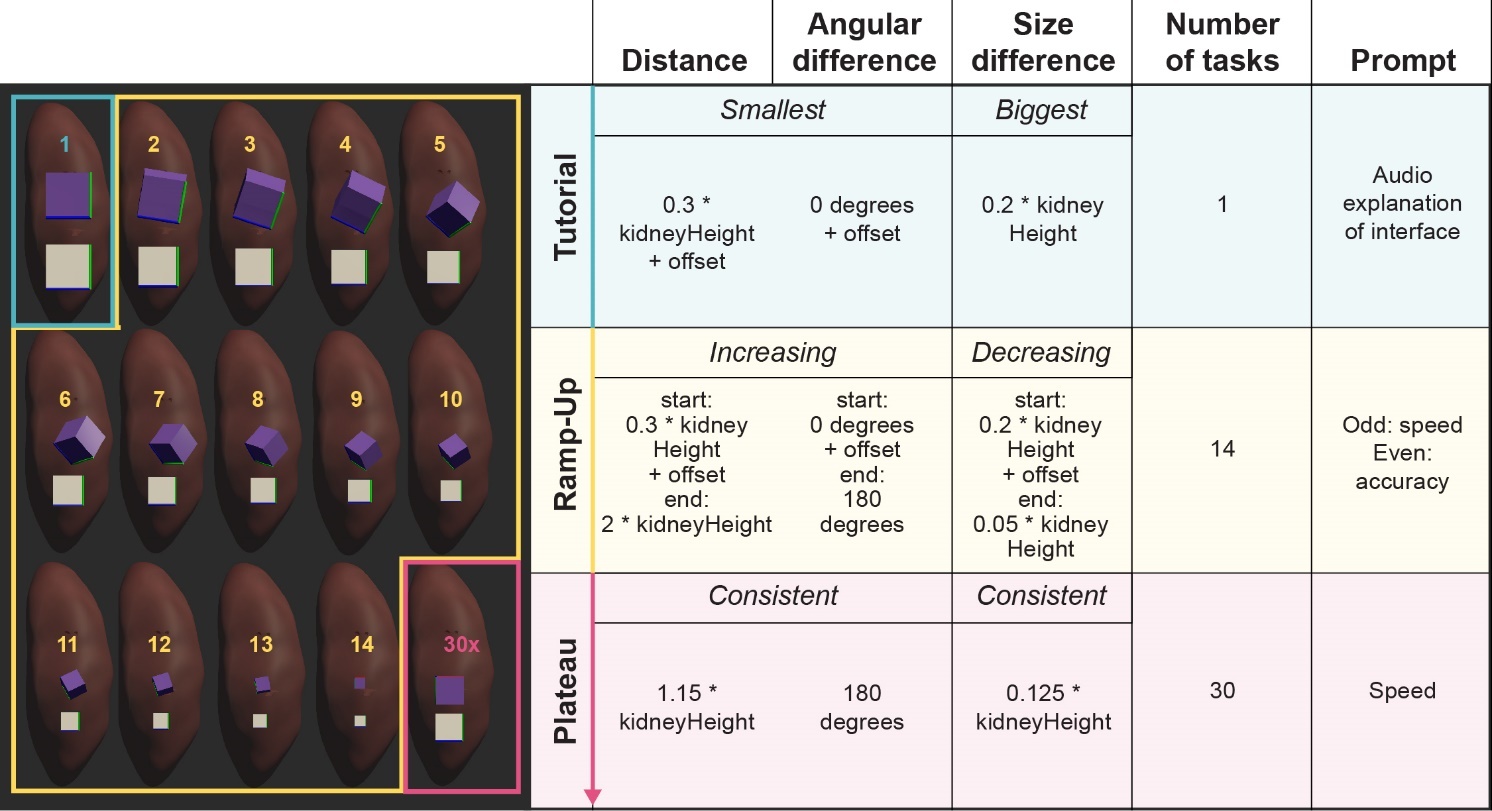


**Supplementary Figure 3. Task setup and levels of difficulty used in this study**. The offset (computed via **Supplementary Equation 1** **above**) is a value that is added to gradually increase the distance and angular difference between the two blocks, and that is used to gradually decrease the size of the two blocks (Bueckle et al., 2021a).

## Analysis of tool usage during Reflective phase

**Supplementary RQ**: In the Reflective phase, how do users apply the interactive tools? 
**Supplementary Ha**: Most users will use the time slider to scroll through around 10 times the time span of their dataset.
**Supplementary Hb**: The most selected location for the play head of the slider will be towards the very end of the timecode in the dataset. 
**Supplementary Hc**: Users will spend the majority of time with the kidney turned on as the presence of a reference organ is highly useful to understand the data overlay. The kidney is turned on by default.

We recorded a variety of metrics from VR subjects in the experiment group during the Reflective phase. This phase consisted of two parts: an intro where the user explored the best-performing user’s dataset (with an audio tutorial about the interactive tools and goals of this phase) and, subsequently, the main part where they explored their own data. Supplementary Table 1 gives the number of observations (N), mean, standard deviation (SD), median, min, and max value for a series of metrics from the intro as well as the main part of the Reflective phase for both VR setups together and separate. Supplementary Table 2 provides definitions of variables for user behavior from the Reflective phase.

On average, users spent **464.63** **seconds** (VR Tabletop, **SD = 149.82 s**) and **396.76 s** (VR Standup, **SD = 268.8 s**) in the main part of the Reflective phase. Unsurprisingly, VR Tabletop users traveled less with their HMDs (**26.88 meters**, **SD = 12.49 m**) than VR Standup users (**41.96 m**, **SD = 19.92 m**). Notice, however, the large **range 114.22 m** between the subject with the **most** **right-hand movement** and the subject with the **least** (both in the VR Standup setup)**.** Likewise, the subject with the most **distance traveled for the HMD** in the VR Standup setup was measured at **81.41 m** (vs. **13.64 m** for the least traveled), yielding a **range of** **67.77 m**.

The bigger freedom of movement probably also to VR Standup users rotating their heads more, with **8434.43 degrees** vs. **7086.07 degrees** for VR Tabletop, equaling around 26 and 20 theoretical complete head rotations, respectively. Further, on average, VR Tabletop users spent **46.9%** of their time in the main part of the Reflective phase without the kidney, compared to **32.2%** for VR Standup users, prompting us to **confirm Hc** (users will spend the majority of time with the kidney turned on). Because VR Tabletop users could rotate the kidney when completing their tasks, kidney rotations were shown in the Reflective phase, which is users may have had more of an incentive to leave the kidney visible. The average number of tasks simultaneously visible was **6.42** (VR Tabletop, **SD = 3.96**) and **6.01** (VR Standup, **SD = 4.16**). Finally, with regards to time slider usage: VR Tabletop users moved the slider **almost twice as much on average** as VR Tabletop users. Specifically, VR Tabletop users scrolled through **5.15** **times** the time span of their dataset, compared to **2.68** **times** for VR Standup. Since both of these values are far off from the 10 times we predicted in **Ha**, we need to **reject Ha**.

Likewise, the mean position of the raw slider on a scale from 0 (first time stamp, beginning of the dataset) to 1 (last time stamp, end of the dataset) was similar for both setups at **0.79** for VR Tabletop and **0.73** for VR Standup users, requiring us to **reject Hb** (predicting that the most selected location for the slider would be towards the very end of the dataset).

## Metrics

To analyze survey and task data, we defined three performance metrics (position accuracy, rotation accuracy, and completion time) as well as a satisfaction score.

### 3D position accuracy

We defined position accuracy as the distance of the centroids of the tissue block and the target block, see light blue arrow in Supplementary Figure 1. We compute the distance at run time using Vector3.Distance(), a static method in Unity that returns the distance between two points in 3D space. The position of both blocks and the centroid distance was collected at 10 Hz (i.e., 10 times each second).

To make use of the various possibilities for scaling in VR, the kidney was displayed in different heights across setups (but always with the same width-to-height-to-depth ratio). Measured from the lowest to the topmost vertex, the kidney in the two VR setups was 0.59 Unity scene units tall. In VR, scene units correspond to physical meters, so the kidney appeared at a height of 590 mm. Similarly, in the 2D Desktop setup, the kidney appeared at a height of 113 mm on the laptop display (see Supplementary Figure 1). In order to compare position accuracy results between 2D Desktop and the VR setups, we normalized these values by dividing them by the height in which the kidney appeared to the user.

### 3D rotation accuracy

Rotation accuracy equals the angular difference between the two tissue blocks at task submission (see Supplementary Figure 1). For ease of analysis, it was reduced to an individual number between 0 (exact same rotation) and 180 (diametrically opposite rotation). We used Unity’s built-in Quaternion.Angle() function to compute this angle. Angle() takes two orientations, each consisting of three angles, expressed either as Euler angles or Quaternions, and returns a single float value between 0 and 180.

This means that several combinations of different rotations between tissue block and target block could yield the same angular difference. In order to preserve as much detail about the subject’s action as possible, equivalent to the position, we logged the rotation of both blocks throughout the experiment as well.

### Completion time

Completion time refers to the amount of time between the submission of a task and the submission of the previous task. Completion time is measured in seconds.

### Performance plateau

During the Plateau phase (**Supplementary Figure 3**), subjects performed 30 identical tasks, providing a unique opportunity to identify if and when a subject achieves a performance plateau. A plateau of a performance variable (task completion time, centroid accuracy, or rotation accuracy) is reached when the deviation of the performance variable does not exceed the mean performance of the subject until the end of the Plateau phase. As mean performance, we consider the average performance in a moving window of 20 tasks of the subject to reduce the influence of possible performance outliers. This width of the moving window supplies a stable mean by considering a certain inertia in performance improvement without including at all times the extreme values that can often be found towards the beginning and the end of the Plateau phase. For each subject, we analyzed after which task the performance stabilized by iterating through a recursive process, in which the relative deviation of the last task of the Plateau phase is calculated. If it does not exceed one (thus if the deviation of the performance variable in this task is not higher than its mean) we iterate this calculation for the previous task until we arrive at a task where the relative deviation is larger than 1. We consider all tasks after this (until the last task of the phase) to be on a performance plateau. If a subject reaches a performance plateau, we take the average performance (for example, mean completion time per task) of all the tasks that are completed after reaching this plateau.

### Satisfaction

To assess user satisfaction, we included a corresponding item in the post-questionnaire via a five-point Likert scale from one (not at all satisfied) to five (very much satisfied), with three being a neutral value, and we report results aggregated by setup.

# Data Availability

We made the raw data generated for our paper available via Zenodo (Bueckle et al., 2021c; b). More information can be found in our GitHub repository for Supplemental Material at <https://github.com/cns-iu/optimizing-performance-in-VR-using-DVL-FW#raw-data>.

# Supplementary Figures


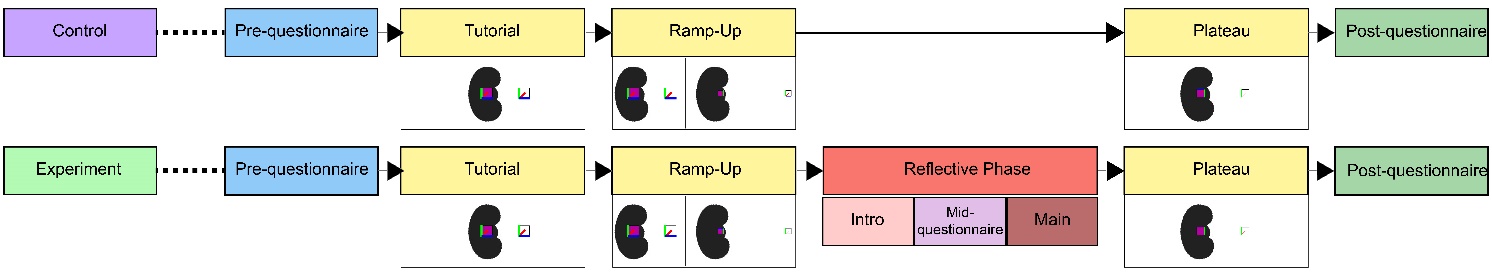


**Supplementary Figure 4.** The study design of the RUI VR user study in detail. Both cohorts filled out a pre-questionnaire before completing a series of VR tasks in three stages: Tutorial (one task, not timed or counted towards performance), Ramp-Up (14 increasingly difficult tasks), and Plateau (30 identical tasks). The experiment cohort participated in a Reflective phase, separated into an Intro part (where they investigated a visualization of the tissue block placements and head/hand movements of the highest performing user in their setup) and a Main part (where they investigated the data from their own Ramp-Up phase. Note that we did not explicitly tell the experiment users that the data in the Intro part came from the highest-performing user. Finally, both cohorts filled out a post-questionnaire to assess satisfaction and gather feedback about the experiment and the setup that the subject was using to complete their tasks (2D Desktop, VR Tabletop, and VR Standup).

**
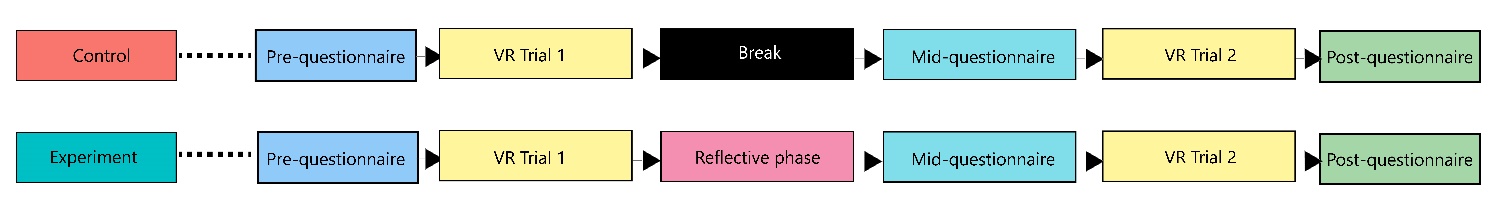
**

**Supplementary Figure 5.** The study design of the Luddy VR user study in detail. Both cohorts started with a pre-questionnaire before completing 24 navigation tasks (including 4 tutorial tasks) during VR Trial 1. The control cohort then took a break, and the experiment cohort investigated their own data in a Reflective phase. Both cohorts then filled out a mid-questionnaire about the virtual building, the number of tasks in total, per floor, etc. Finally, all subjects completed a post-questionnaire to assess satisfaction and gather feedback about the experiment.


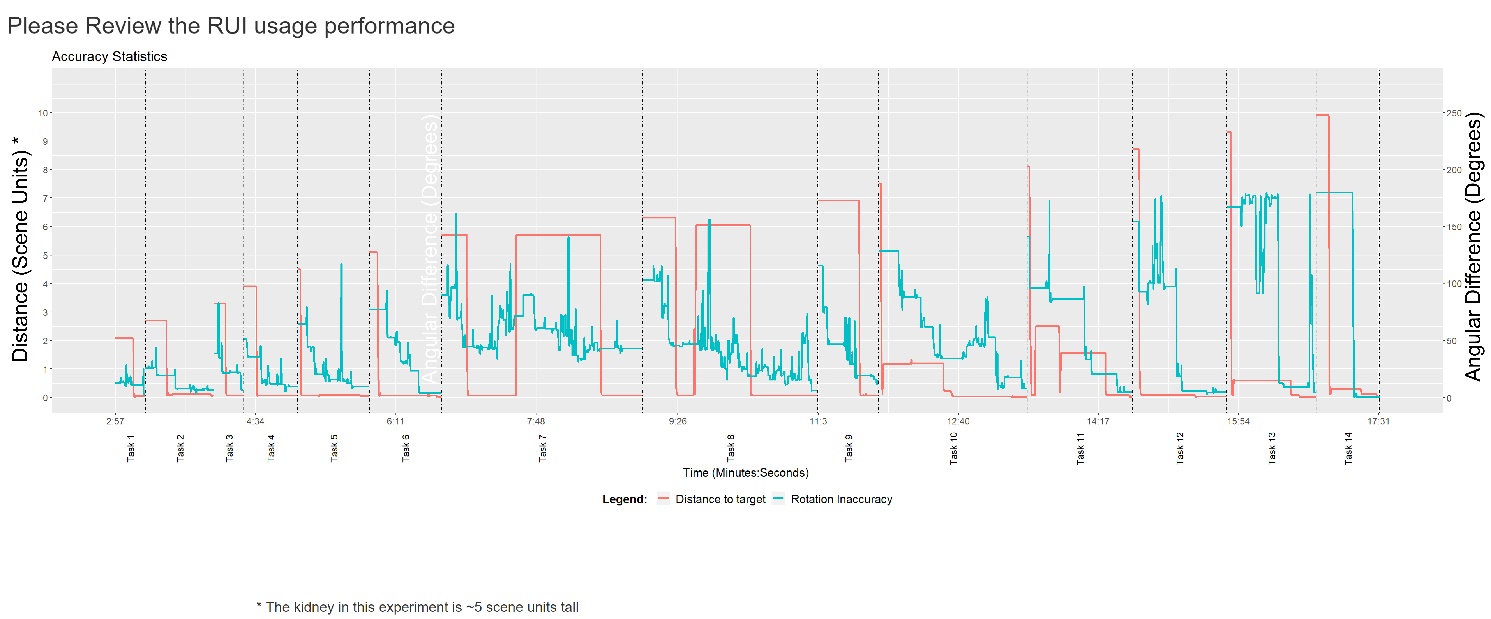


Supplementary Figure 6. Line graph of distance between tissue and target block (orange) and angular difference (green) for the best user in the control cohort for 2D Desktop.


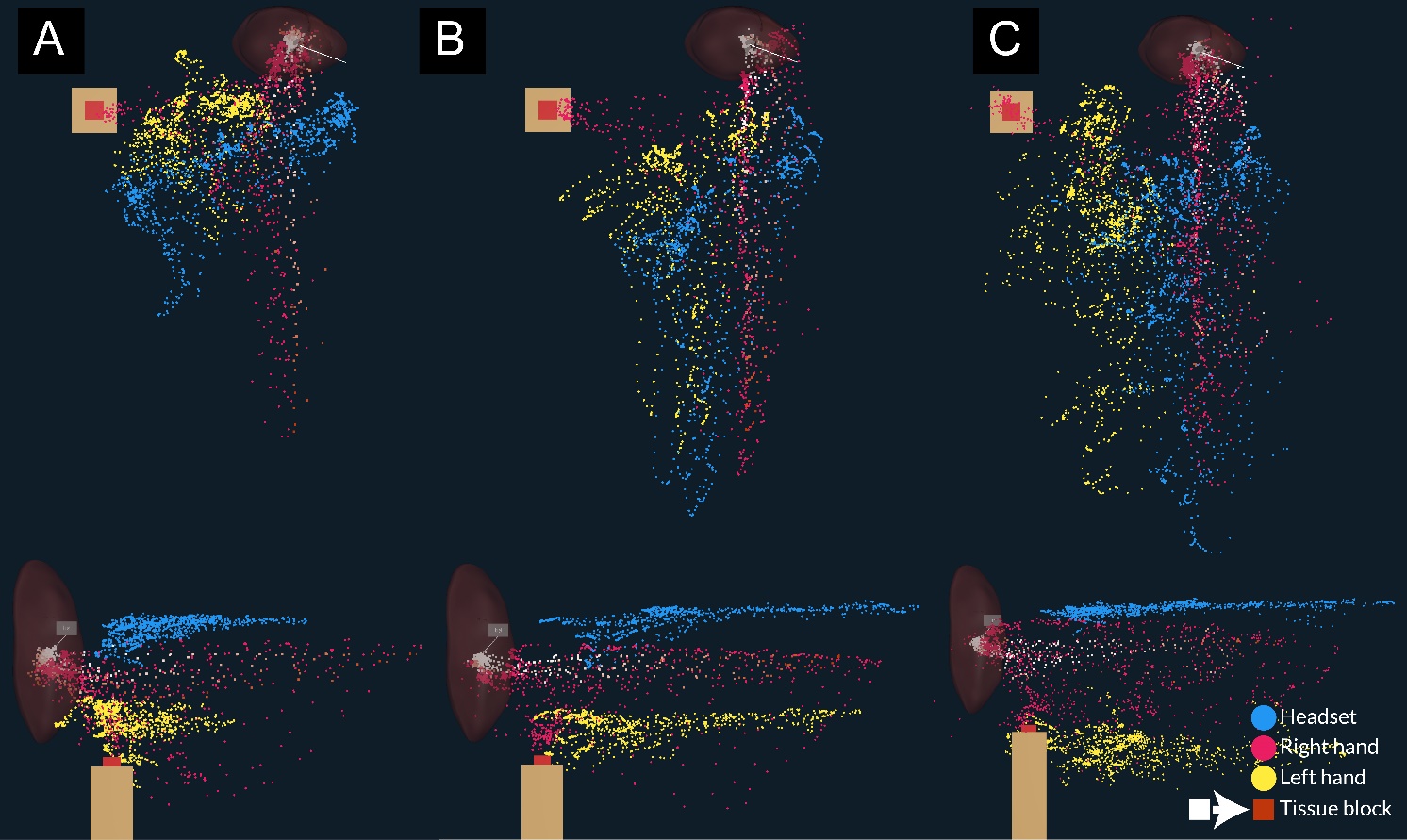


Supplementary Figure 7. Top view visualizations for three subjects with various spatial usage patterns (Standup). Left: concentrated work on one axis (see HMD in blue). Middle: plenty of back-and-forth along the z-axis. Right: wide spread around the z and x-axis.


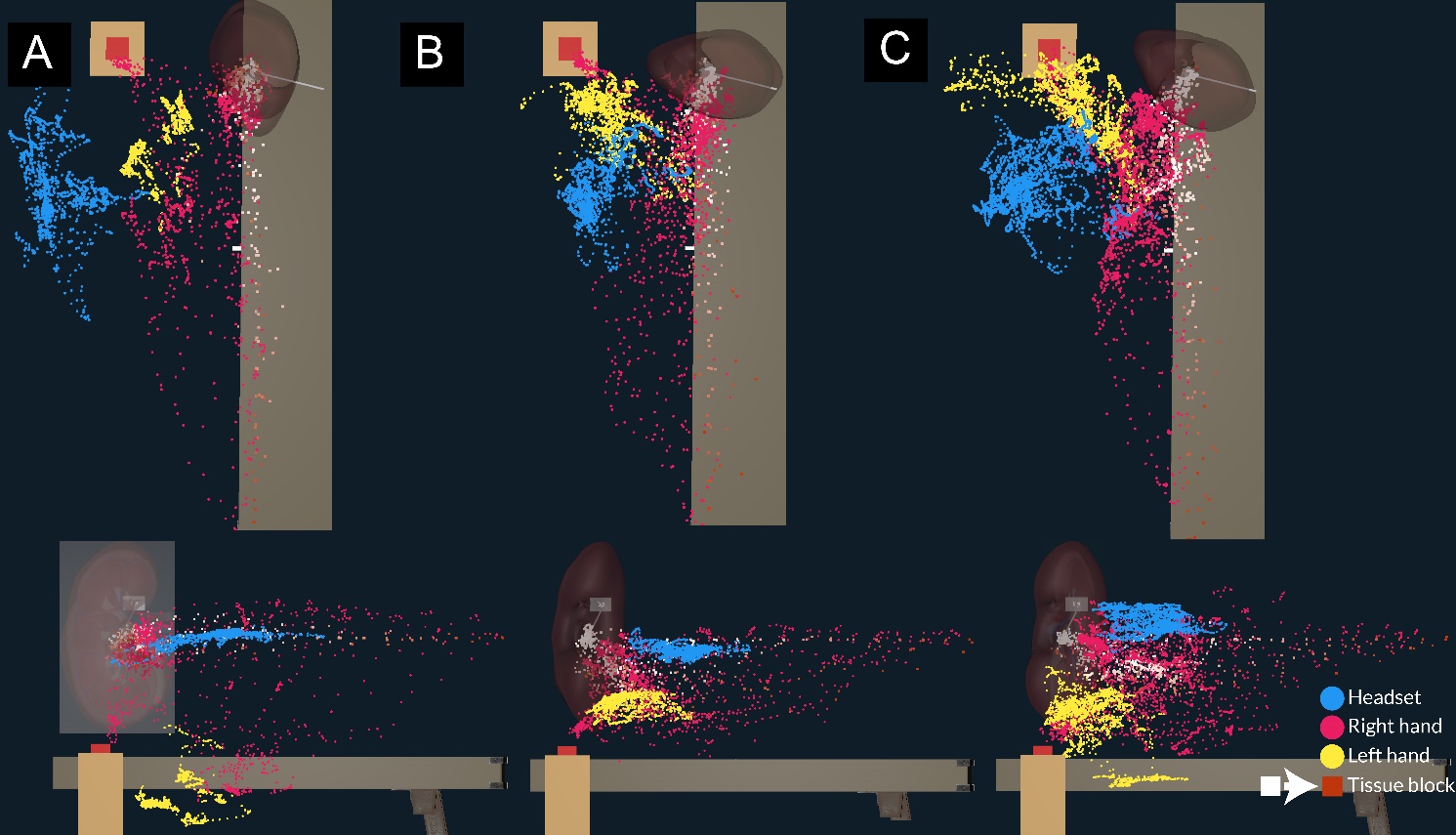


Supplementary Figure 8. Top view visualizations for three subjects with various spatial usage patterns (Tabletop). A: concentrated work on one axis. Middle: plenty of back-and-forth along the z-axis. Right: wide spread around the z and x-axis.


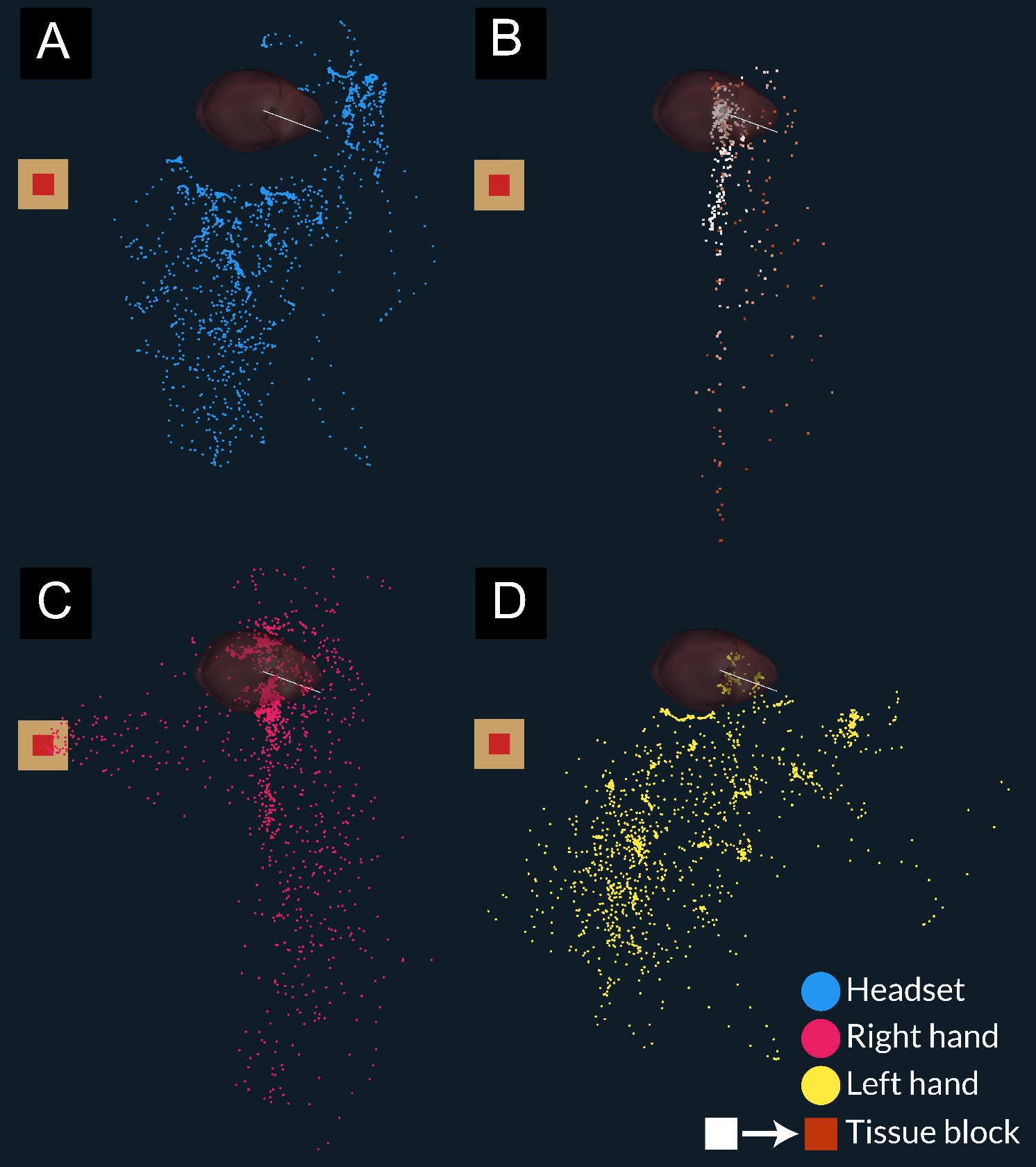


Supplementary Figure 9. Data overlay for the VR setups separated by color. A: HMD. B: tissue block. C: right controller. D: left controller.


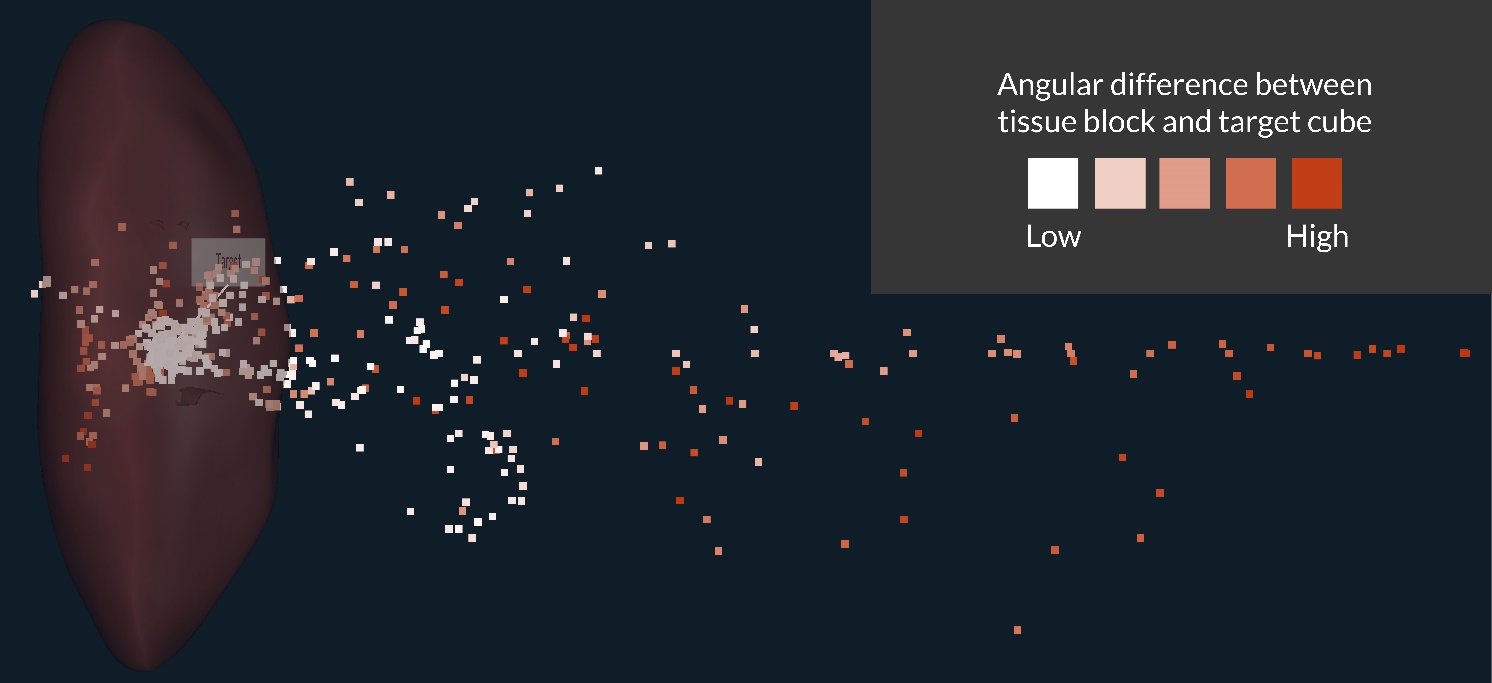


Supplementary Figure 10. Distribution of tissue block locations over time, with angular difference between the tissue and target block encoded with a sequential color scheme.


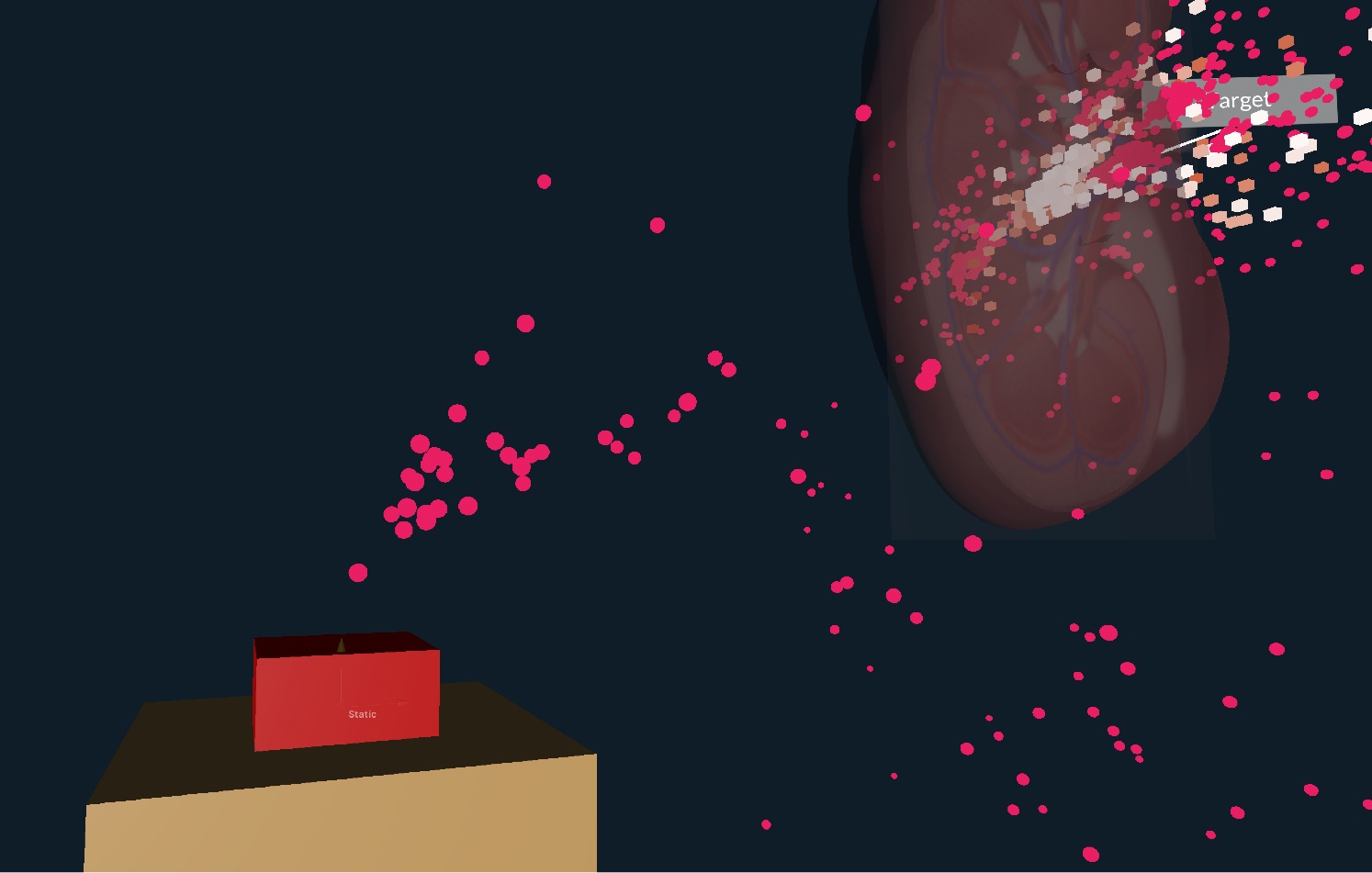


Supplementary Figure 11. The user's repeated pressing of the virtual red buzzer produces a hot spot.


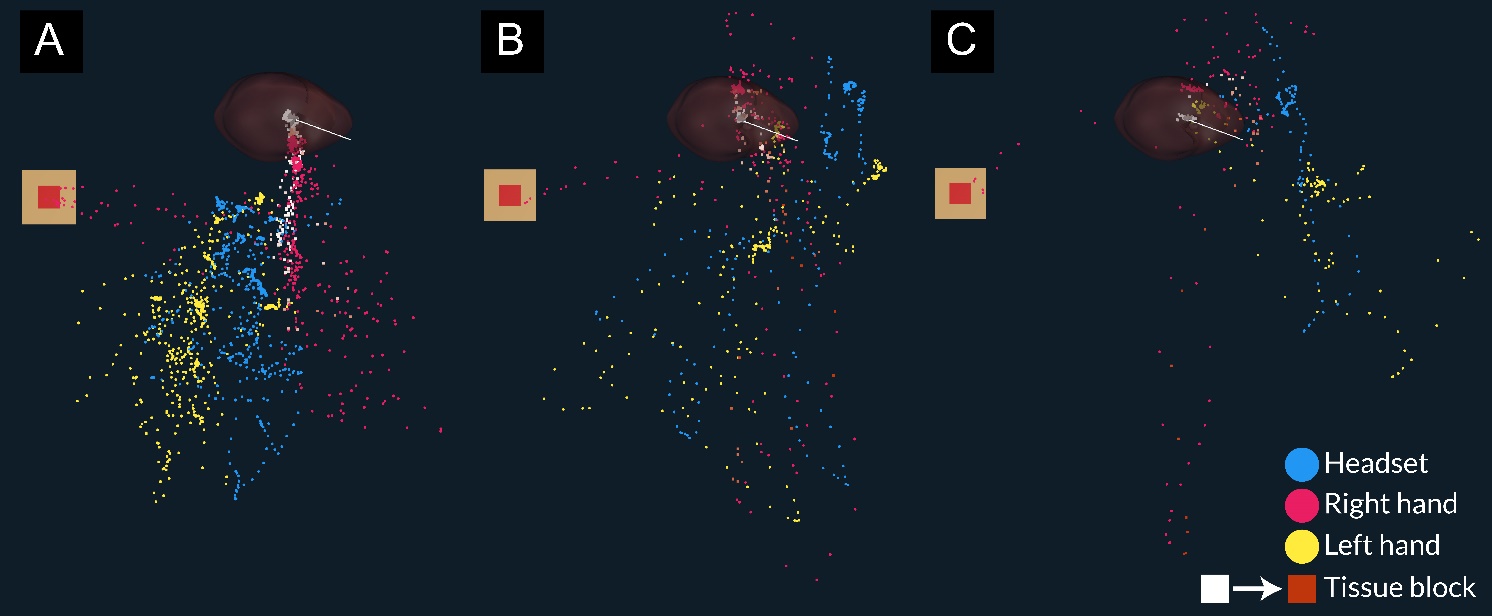


Supplementary Figure 12. VR Standup user with three different stages shown. A: Tasks 1-5. B: Tasks 10-11. C: Only task 14


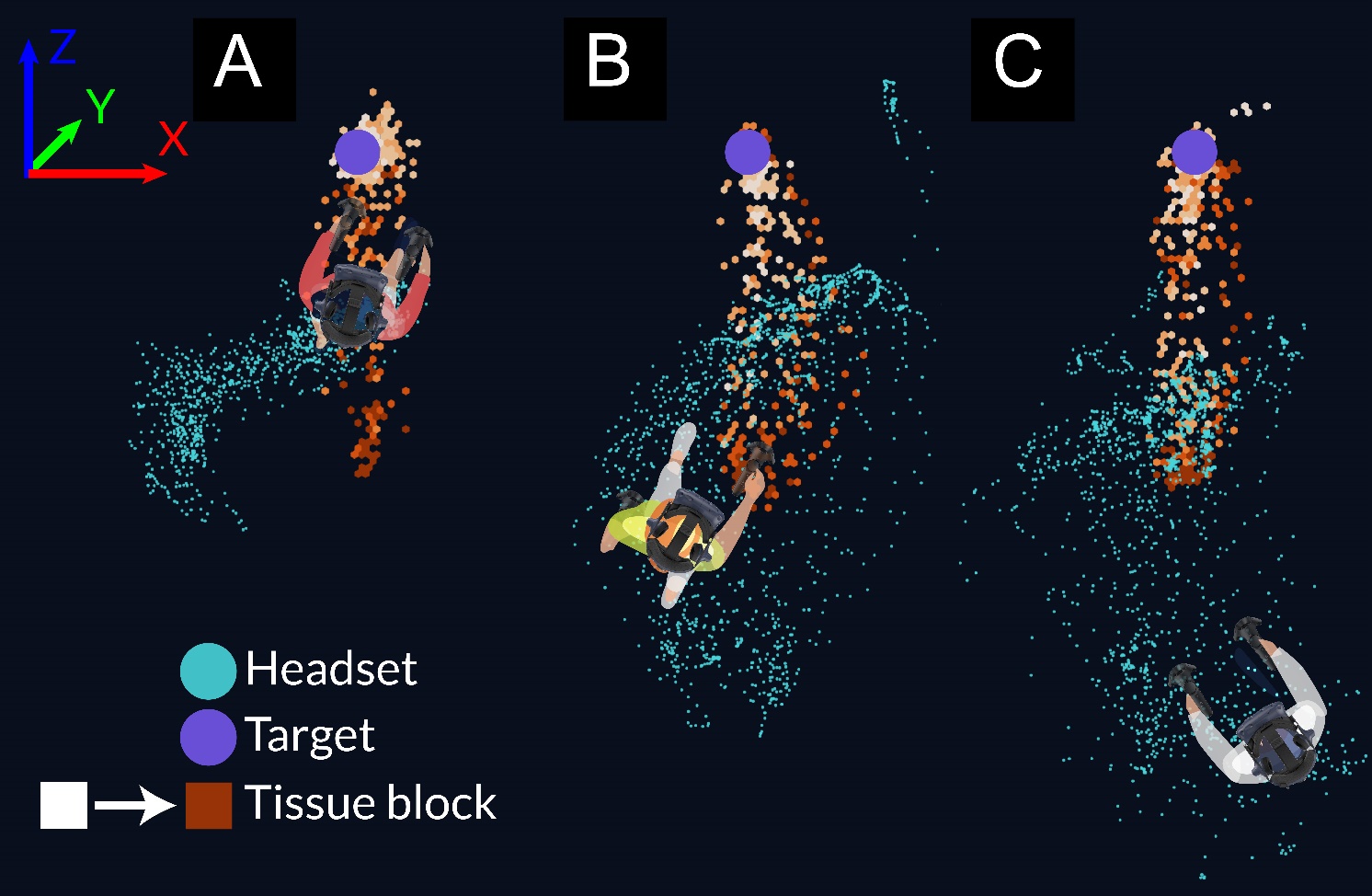


Supplementary Figure 13. Overhead view (x-z plane) of three participants (A, B, C) with unique movement patterns during the Ramp-Up phase.


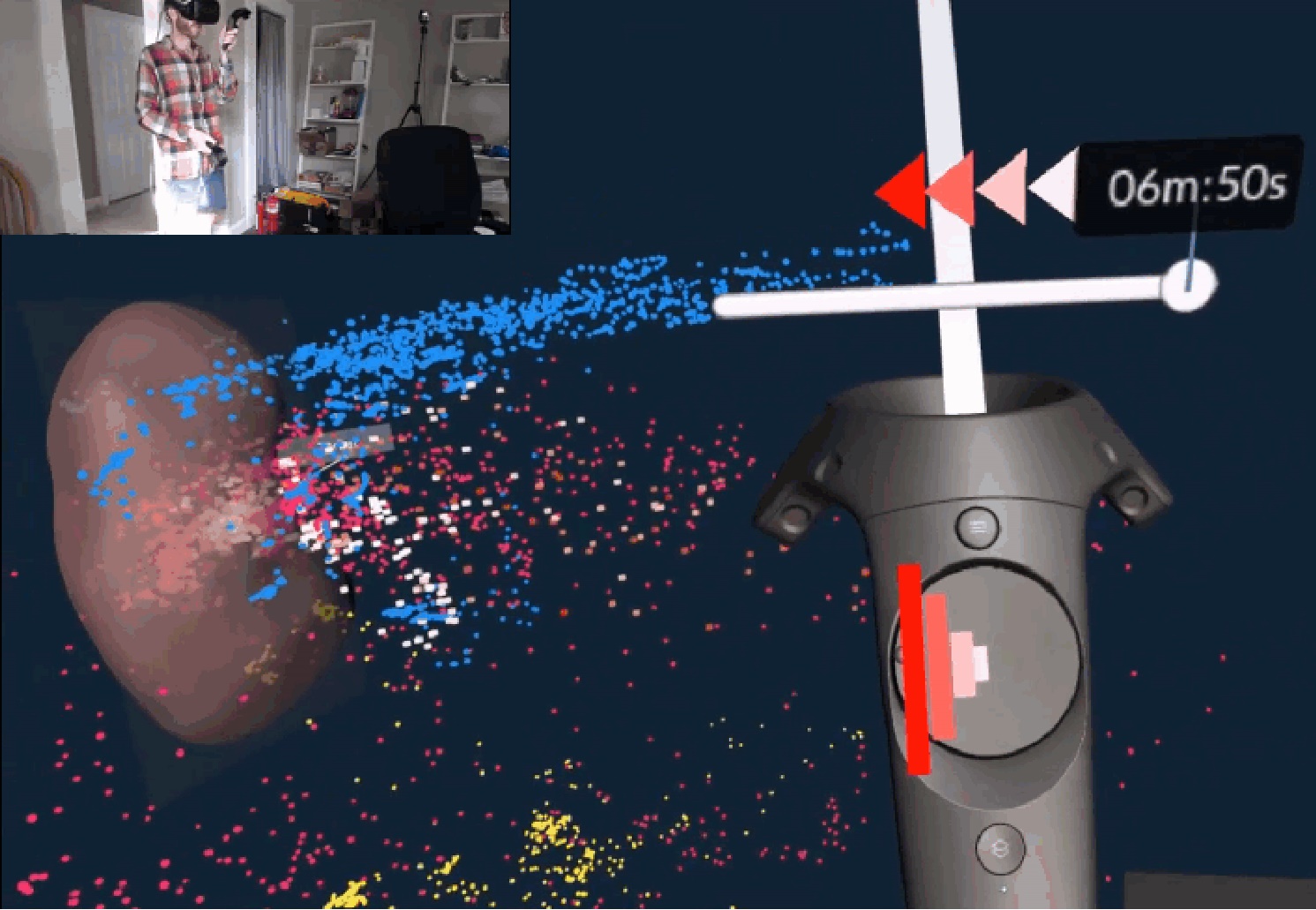


Supplementary Figure 14. Time slider to skip forward and backward in time by using via the thumbpad on the VR controller.


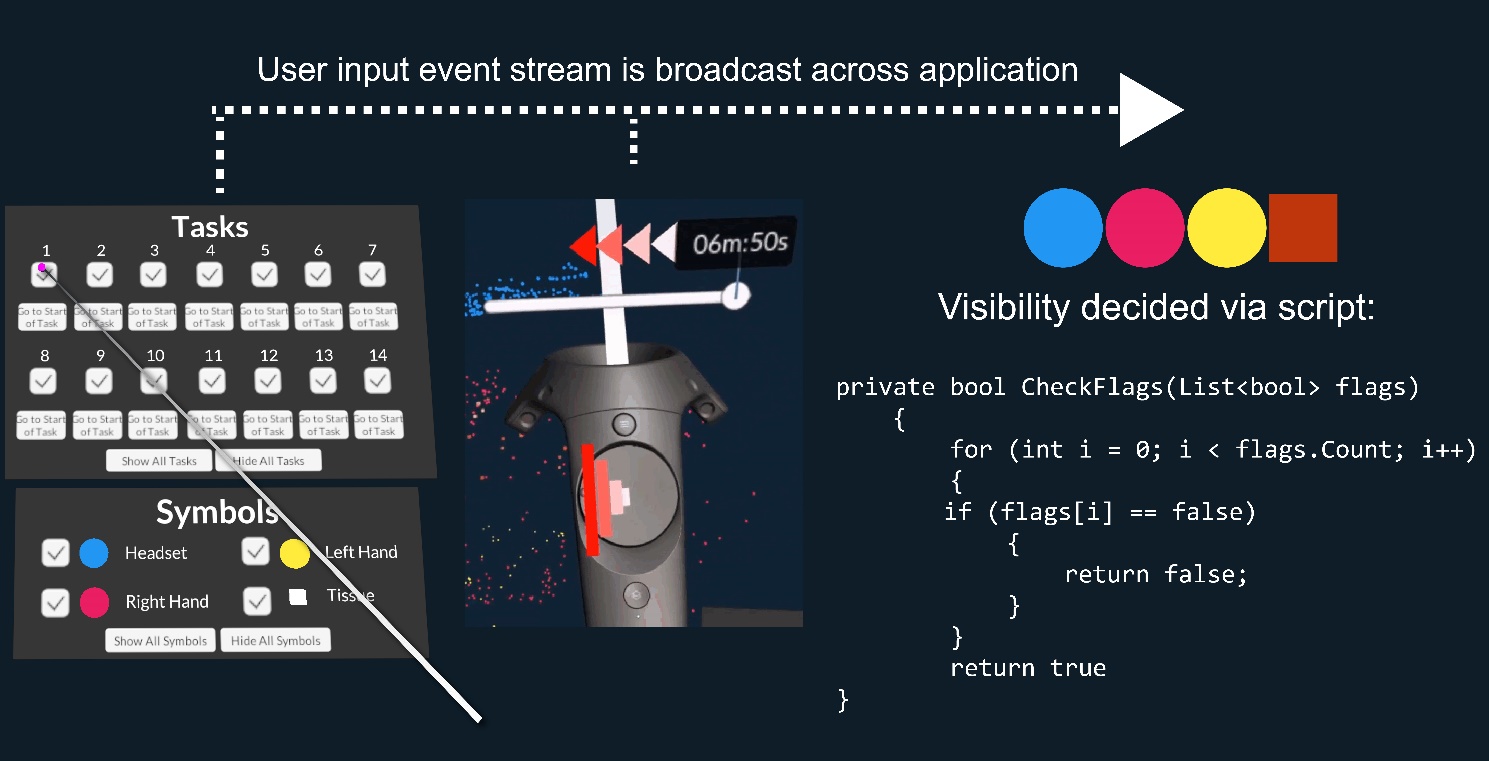


Supplementary Figure 15. Abstract image of the event listener for each graphic symbol. A series of Boolean values is passed into the CheckFlags() function, for example, whether the currently shown time stamp is later than the associated time stamp of the graphic symbol. If all arguments evaluated to true, the graphic symbol was displayed.


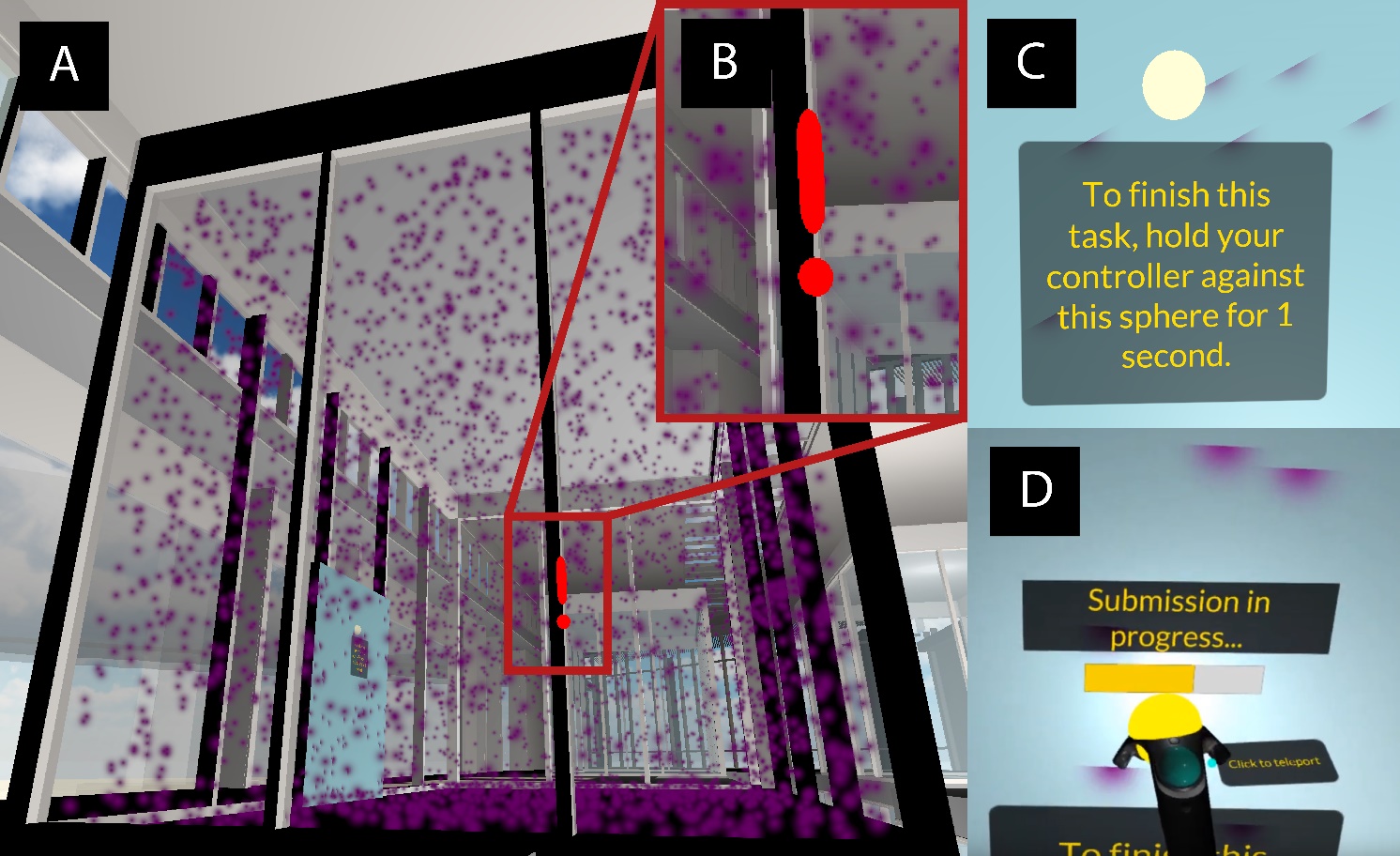


Supplementary Figure 16. Anatomy of a task room. A: the tutorial task room from the outside. B: note that the red exclamation mark is rendered on top of the wall in front of it. C: task submission instructions. D: the submission in progress.


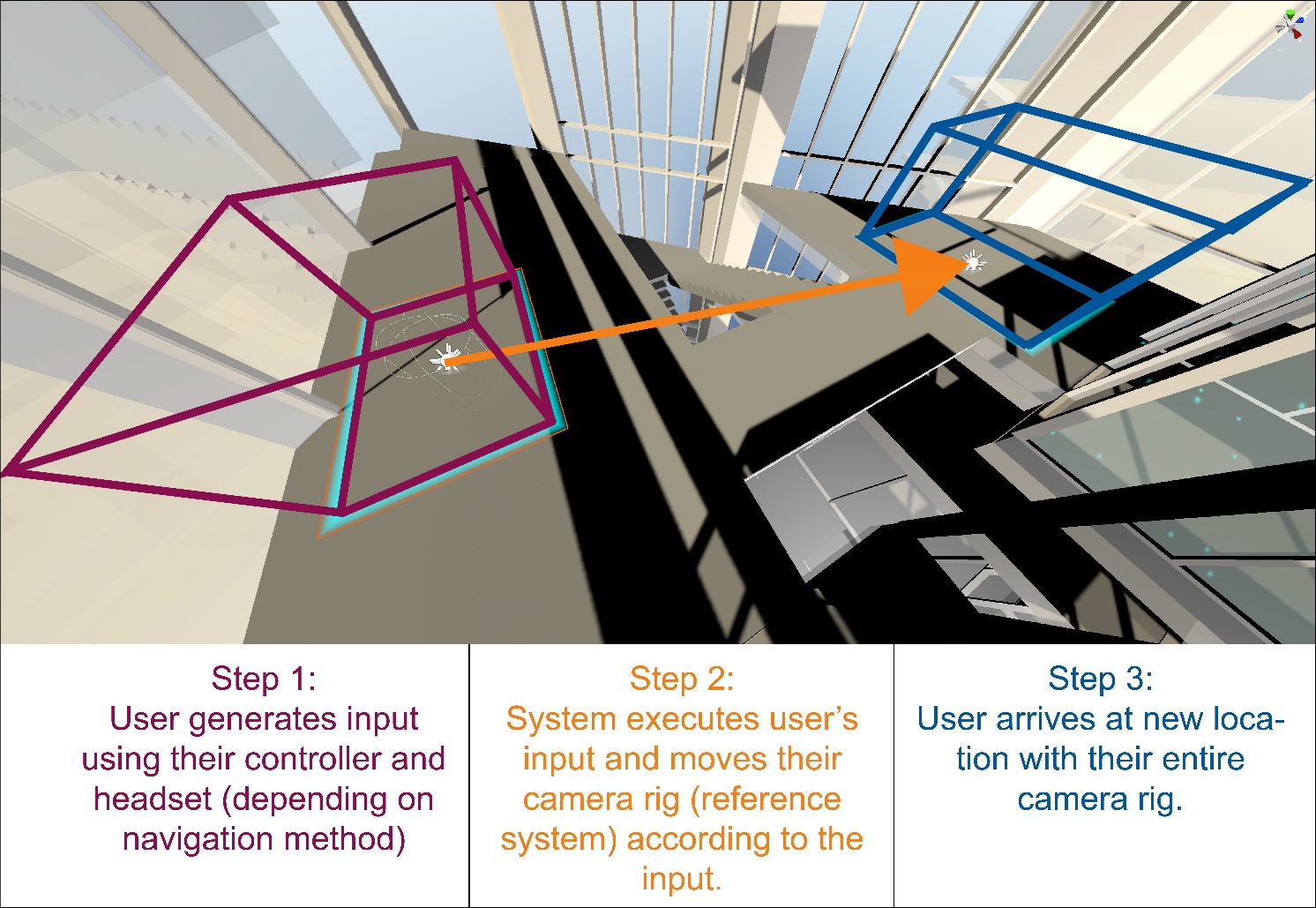


Supplementary Figure 17. Illustration of basic navigation.


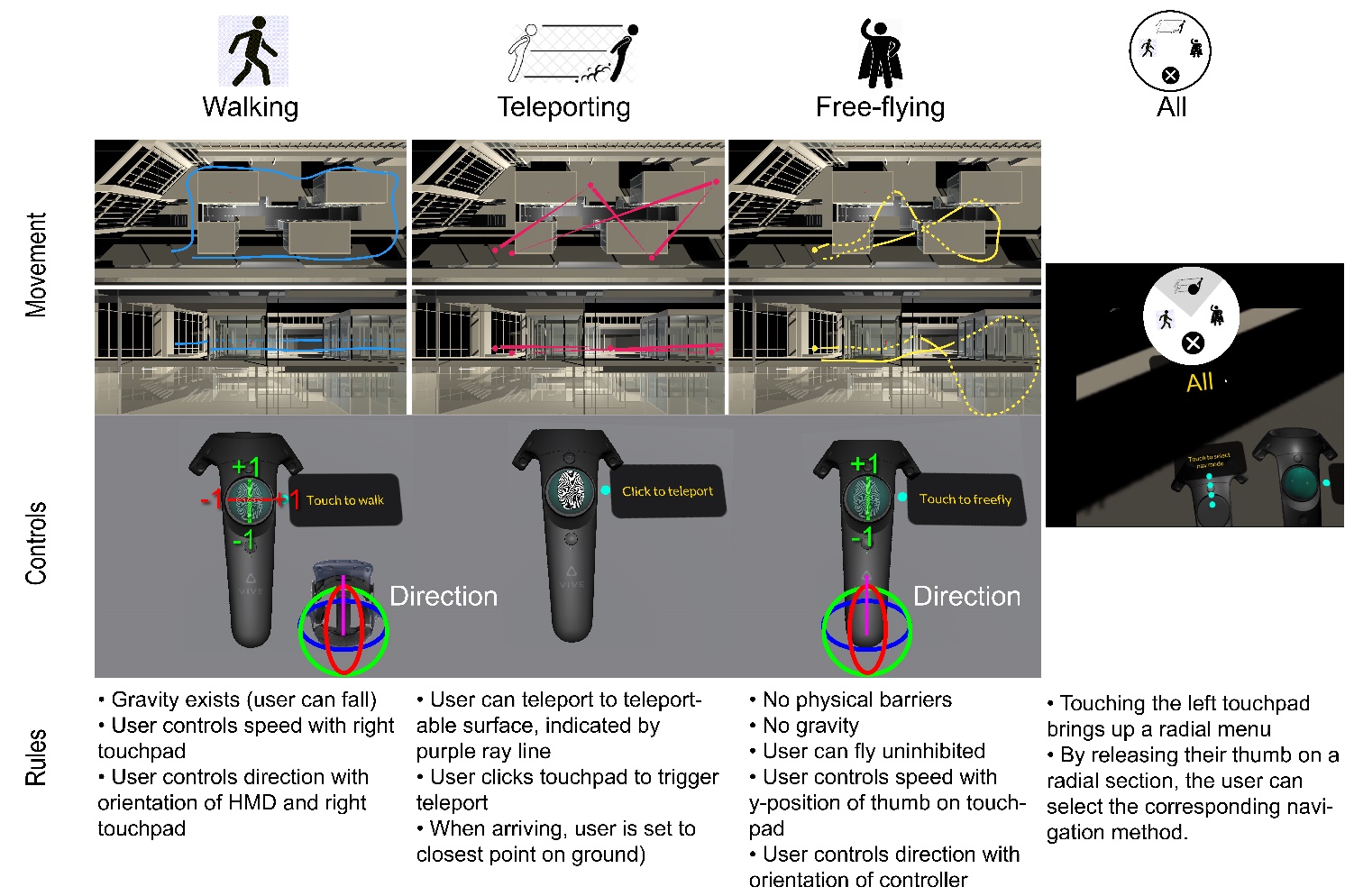


Supplementary Figure 18. A comparison of movement, controls, and features for all three navigation methods.


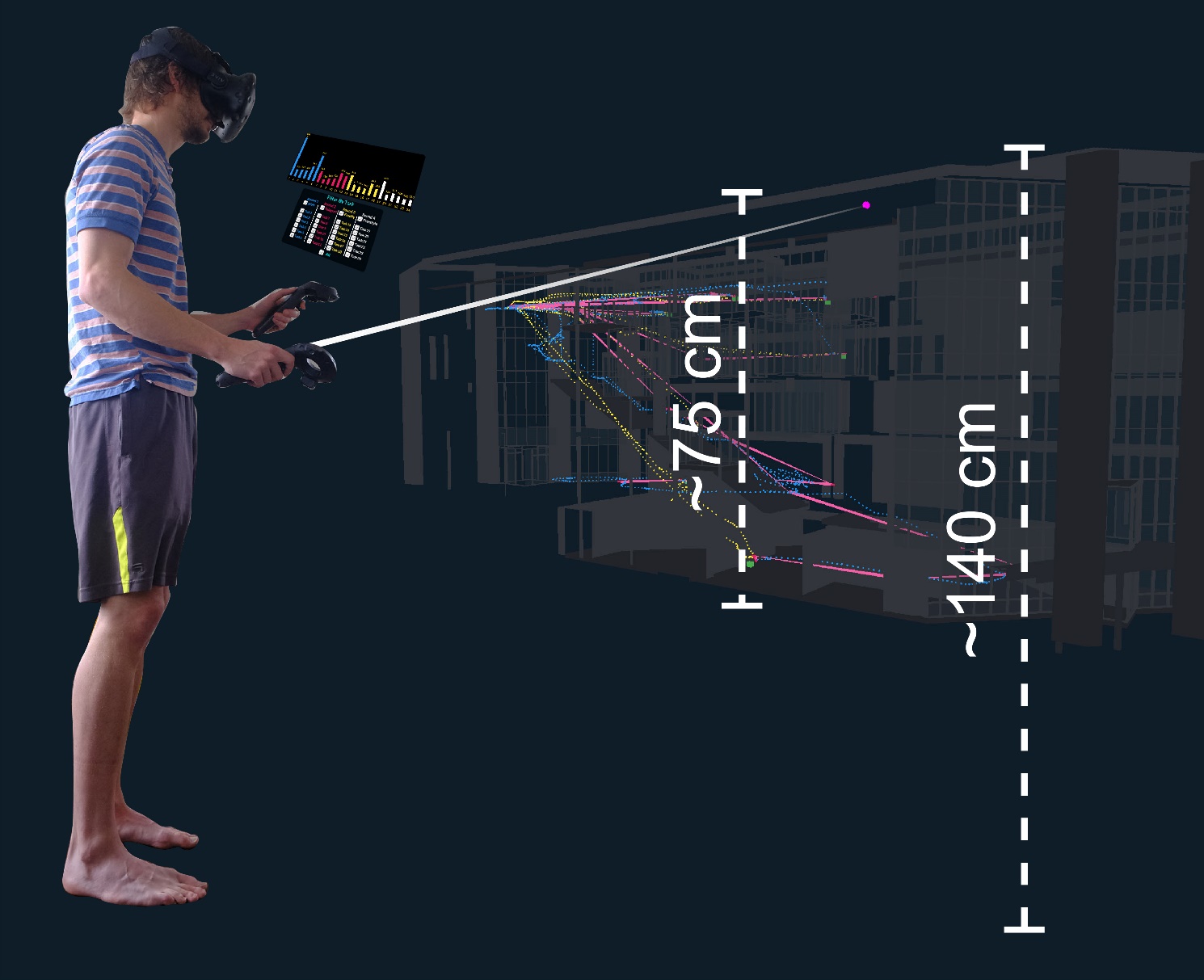


Supplementary Figure 19. Approximation of a user in relation to the 3D base map and visualization in VR.


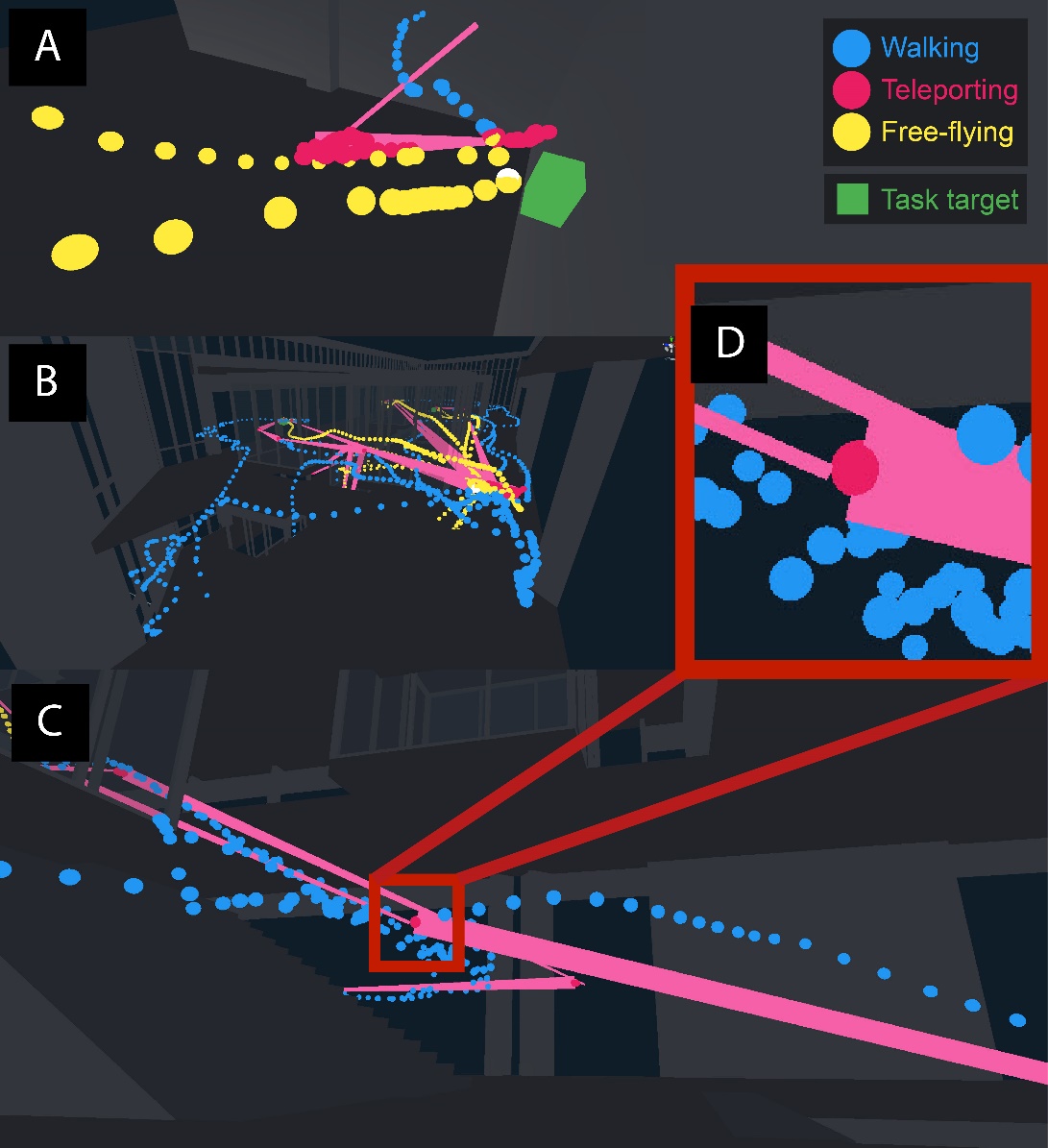


Supplementary Figure 20. Visual encoding details. A: a user’s approaches to task #5. Note that there are two yellow trajectories, because they also used free-fly to finish this task when they had the choice. B: the start position on the fourth floor and the 24 trajectories leading away from it. C: a stop during a series of teleports. D: Note how the line leading to the teleport stop is thin, and the line leading away is wide, signaling the direction of the teleport.


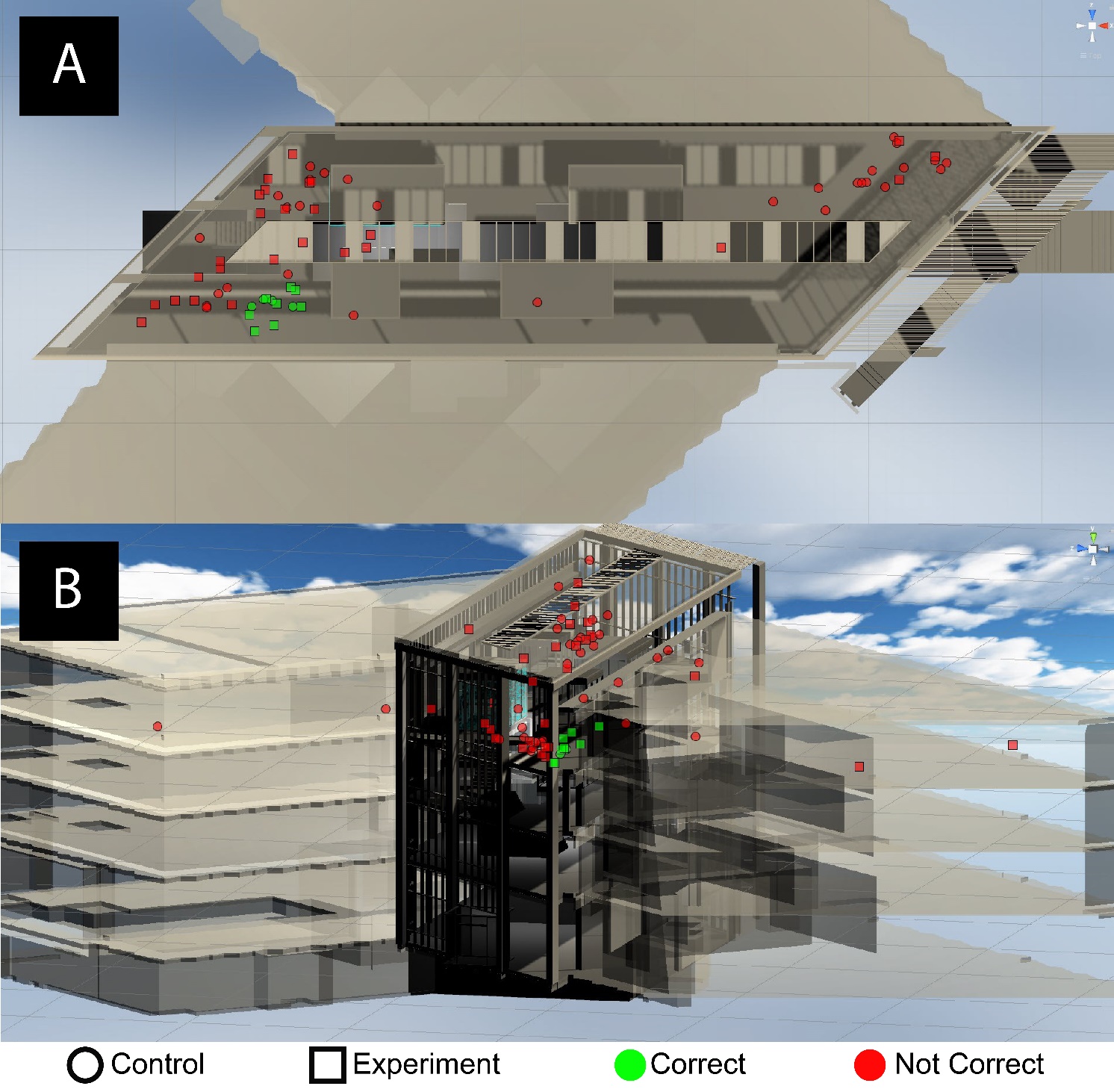


Supplementary Figure 21. Dot density maps pf click positions where users were asked to indicate where in these screenshots their start position was. A: top view. B: side view.

Like the rest of the mid-questionnaire, the visualization shows a stark difference between the cohorts. For the top view, there were 12 correct answers (4 for control, 8 for experiment); for the side view, there were only 10 correct answers (2 for control, 8 for experiment). The difference in correct answers for the side view between the cohorts for this particular task is significant (***t* = -2.0899, *p* = 0.04158**). This is likely thanks to the Reflective Phase, because every trajectory in the Reflective Phase visualization marked the user’s start position (see Supplementary Figure 20 B), thus giving an advantage to the experiment cohort.

While many users were at least able to determine that they had started somewhere in the atrium of Luddy Hall, a surprisingly large number of subjects mistook the barely modeled wings of the building for the location where they spent the experiment (see Supplementary Figure 21B). As becomes apparent from Supplementary Figure 21A, on the other, the top view helped users narrow down the amount of choices, and the majority of subjects picked one of the four corners of the atrium. Note that we found **no significant correlation** between how familiar users were with Luddy Hall as indicated on a 5-point Likert scale and the total score in the mid-questionnaire.


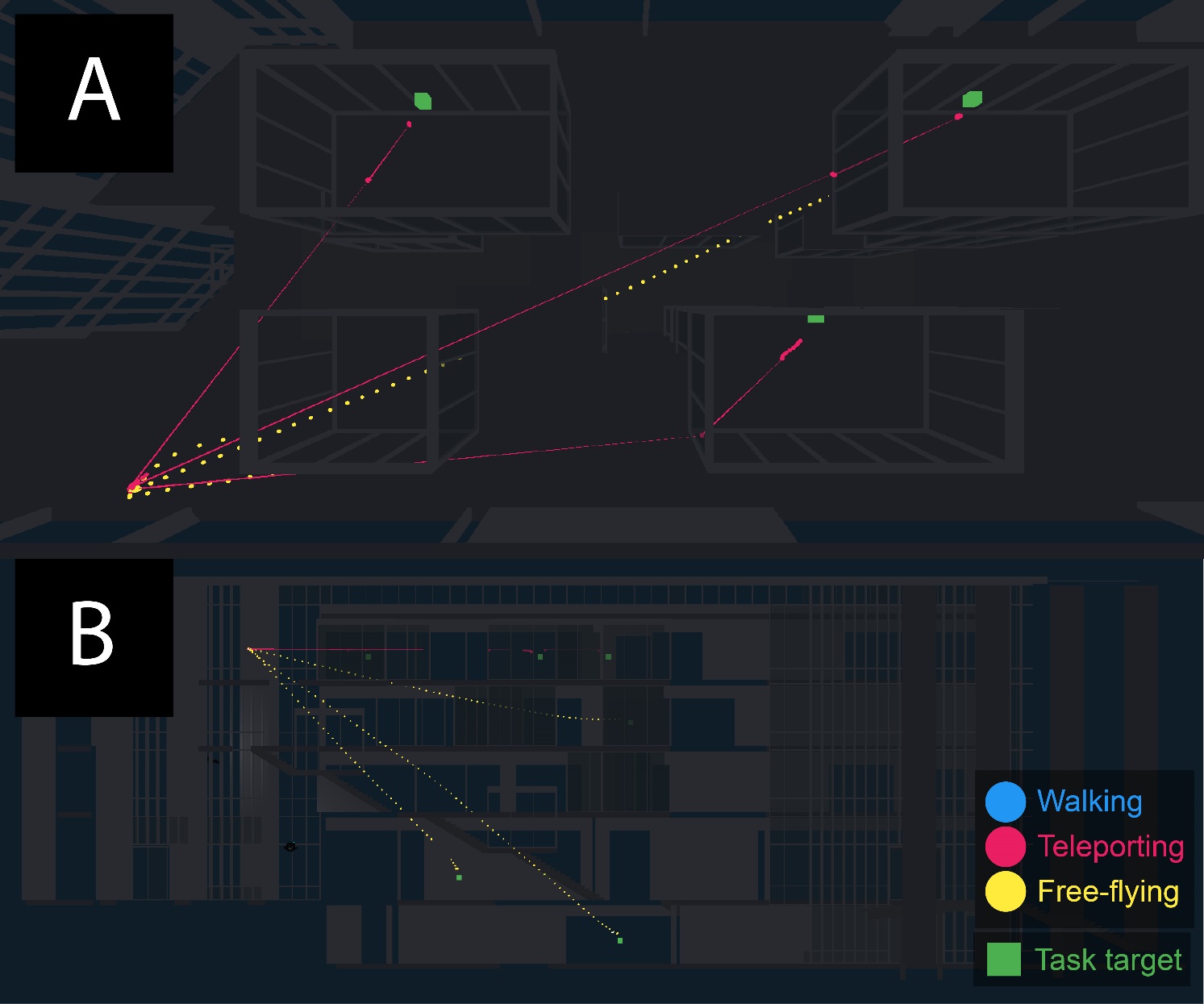


Supplementary Figure 22. The "winning strategy". A: using teleport for targets on the same floor as the start position. B: using free-fly for all others.


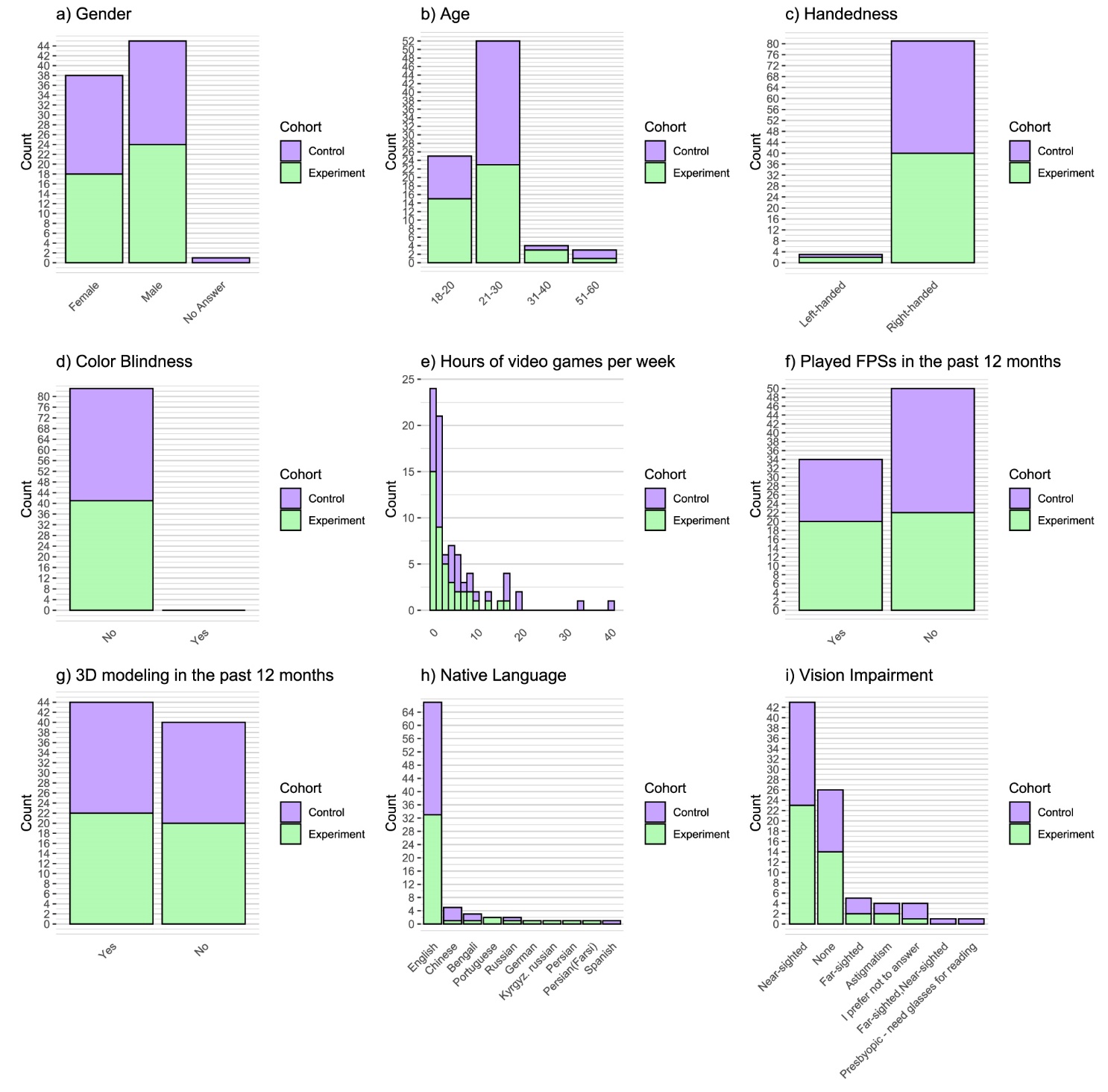


Figure 23. Demographic make-up of RUI VR participants.

# Supplementary Tables

Supplementary Table 1. Summary statistics of tool usage during Reflective phase.

|  | **VR Tabletop** | **VR Standup** |
| --- | --- | --- |
| **Mean time spent** | 464.63 s (~7.7 mins) | 396.76 s (~6.6 mins) |
| **Mean distance traveled** | 26.88 m | 41.96 m (min = 13.635 m, max = 81.413) |
| **Mean total head rotation (y-axis)** | 7086.07 degrees (39.4 left-to-right head rotations) | 8434.43 degrees (46.9 left-to-right head rotations) |
| **Mean number of visible tasks** | 6.42 | 6.01 |
| **Times scrolled through length of dataset (H3a** 🗶**)** | 5.15 | 2.68 |
| **Mean time spent without kidney (H3c ✓)** | 46.9% | 32.2% |

Supplementary Table 2. Definition of variables for user behavior from the Reflective phase.

| **Variable** | **Definition** |
| --- | --- |
| **total_time_spent** | time spent in the reflection phases |
| **distance_[INPUT DEVICE** | cumulated movement of left hand, right hand and head (in meters) |
| **degree_headrotationY** | Cumulated total degrees of head rotation around the y-axis |
| **head_upDownY** | Cumulated total head movement up and down the y-axis |
| **mean_rawSlider** | Average raw slider position ranging from 0 to 1 for each subject |
| **amountKidneyTurnoff** | Total number of times the kidney visualization was turned off |
| **time_without_kidney** | Share of reflective time spent with kidney turned off |
| **time_toggle_filter_usage** | Share of reflective time spent with other filter toggles used |
| **avg_task_visible** | Average of task numbers that were visible during reflective phase |
| **avg_number_tasks_visible** | Average numbers of tasks that were visible at the same time |

All the values reported below have been recorded in the **main** part of the Reflective phase.

Supplementary Table 3. The p-values of the Kruskal-Wallis-Tests for left-handed/right-handed subjects and their performance (no significance). Note there were only 3 left-handed subjects. The column for VR Standup is empty, because there were only right-handed people in this setup.

|  | **Setup** | | |
| --- | --- | --- | --- |
| **Performance metric** | **2D Desktop** | **VR Tabletop** | **VR Standup** |
| **Completion time** | 0.142857142857143 | 0.428571428571429 | N/A |
| **Position accuracy** | 0.785714285714286 | 0.761904761904762 | N/A |
| **Rotation accuracy** | 1 | 0.64021164021164 | N/A |
| **Satisfaction** | 0.136345217441964 | 0.956806206691767 | N/A |

# References

Bueckle, A., Buehling, K., Shih, P.C., and Börner, K. (2021a). 3D virtual reality vs. 2D desktop registration user interface comparison. *PLOS ONE* 16(10)**,** e0258103. doi: 10.1371/journal.pone.0258103

Bueckle, A., Buehling, K., Shih, P.C., and Börner, K. Data from 3D Virtual Reality vs. 2D Desktop Registration User Interface Comparison*.* Zenodo. (2021b) <https://doi.org/10.5281/zenodo.5189516>.

Bueckle, A., Buehling, K., Shih, P.C., and Börner, K. Data from Optimizing Performance and Satisfaction in Matching and Movement Tasks in Virtual Reality with Interventions Using the Data Visualization Literacy Framework*.* Zenodo. (2021c) <https://doi.org/10.5281/zenodo.5658725>.

Unity Technologies (2021). *Unity real-time development platform* [Online]. Available: <https://unity.com/> [Accessed 12/03/2021].
